# Market-driven, value-based, advance commitment (MVAC): accelerating the development of a pathbreaking universal drug regimen to end TB

**DOI:** 10.1136/bmjgh-2019-002061

**Published:** 2020-04-23

**Authors:** Kalipso Chalkidou, Adrian Towse, Rachel Silverman, Martina Garau, Ganesh Ramakrishnan

**Affiliations:** 1 Center of Global Development, Washington, District of Columbia, USA; 2 Global Health and Development Group, Imperial College London, London, United Kingdom; 3 Office of Health Economics, London, UK

**Keywords:** tuberculosis, health economics, health systems evaluation, infections, diseases, disorders, injuries, health policies and all other topics

**Summary box**

- Tuberculosis (TB) is the world’s deadliest infectious disease; without a significant technological breakthrough, current trajectories suggest that the world will not achieve the Convergence 2035 targets for TB until 2074, almost 40 years later than originally projected.
- Research and development investments for TB are dominated by public sources and total only one-third of estimated need, with private investment small and declining.
- To crowd in private investment, we suggest a new model—the market-driven, value-based advance commitment (MVAC)—wherein high-burden middle-income countries (MICs) would offer advanced purchase commitments for a prespecified breakthrough treatment regimen.
- Through use of early health technology assessment, an emerging practice and capability in many large MICs, the MVAC would ensure that country purchase commitments reflect local needs, value and ability to pay for innovation.
- A multilateral development bank would underwrite the MVAC commitments, increasing their credibility to private industry without requiring countries to put aside funds in advance.
- Discussion with developing country policymakers, industry, development banks and development partners suggest fertile ground for the MVAC approach, but high-level political commitment is still needed.

## Chronic underinvestment in R&D threatens the tuberculosis response

**Figure 1:**
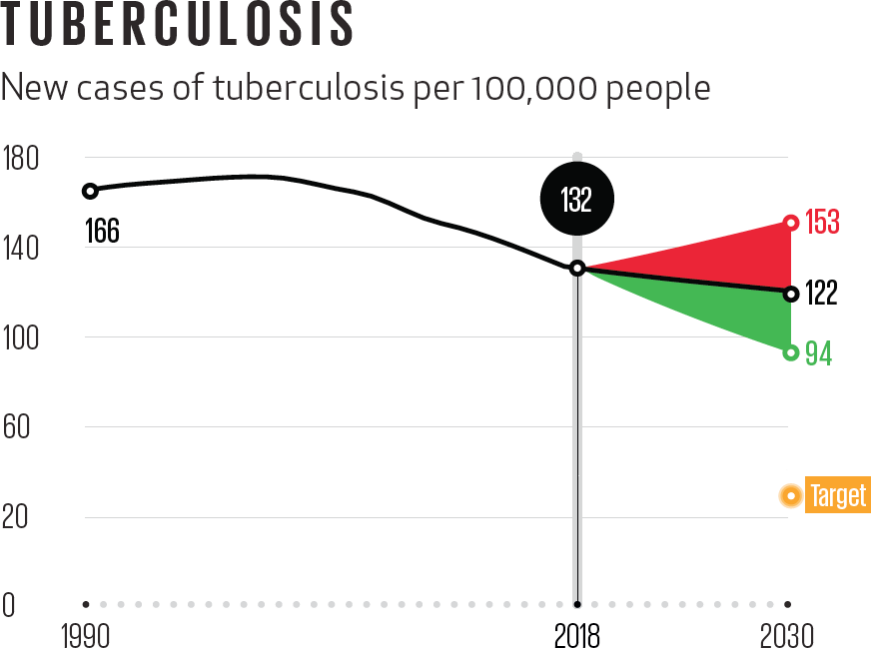
New cases of tuberculosis per 100 000 people. Source IHME. BRICS, Brazil, Russia, India, China and South Africa; HTA, health technology assessment; LMICs, low- and middle-income countries; MICs, middle-income countries.^24^

Tuberculosis (TB), an infectious disease primarily affecting poor and neglected populations, kills an estimated 1.6 million men, women and children each year).^1^ Our existing arsenal of tools is insufficient to address this enormous burden. Current treatment cycles are long and toxic, causing some patients to discontinue treatment, acquire drug resistance and spread a drug-resistant pathogen to others. Treating drug-resistant cases takes even longer, is more expensive and less effective. More extensive forms of drug resistance are developing and spreading quickly. On the current trajectory, the world will not achieve the Convergence 2035 targets for TB until 2074, almost 40 years later than originally projected (see figure 1).

New diagnostics, drugs and vaccines, alongside stronger healthcare systems, are badly needed. All require investment—an estimated $2 billion per year to drive the requisite technological innovation.^2^ Estimates for current research and development (R&D) investments reveal a large funding gap, with total investments of between just $615 million^3^ and $906 million^4^ per year (in 2018), and overwhelmingly donor financed. More than two-thirds were provided by the public sector ($617 million), with the US government financing around 60% of this ($371 million). The remaining public sources are split between the UK (the second largest contributor), the European Union, India, Germany, Canada and South Korea. Private industry accounts for only 9% of total TB R&D funding ($85 million in 2018)—accounting for less than 0.05% of total private-sector pharmaceutical R&D investment and declining.^5^ Philanthropic funding totalled $159 million in 2018, with almost 90% provided by the Gates Foundation. Finally, 5% of total TB R&D in 2018 was given by multilateral funding, mainly financed by Unitaid.^4^

The resulting funding shortfall (over $1 billion) is unlikely to be filled by donors and philanthropists alone. Who will step up and pay for new TB treatments?

## Emerging economies: epicentre of the TB epidemic, source of a transformational solution?

Most TB patients live in fast-growing, emerging economies. Many of these countries have already lost eligibility for traditional donor aid or are currently mid-transition and just five middle-income countries (MICs)—India, China, Indonesia, Russia and South Africa—account for over 40 per cent of TB cases and 55 per cent of cases with multi-drug resistance. They will get the greatest benefit from new TB treatments. Could the most-affected countries also take the lead in shaping an innovation agenda?

There are some positive political signals. The 2018 United Nations high-level meeting on TB^6^ indicated global momentum to step up the fight. TB featured prominently^7^ in discussions and communiqués^8^ at the annual Brazil, Russia, India, China and South Africa (BRICS) summits;^9 10^ the BRICS also launched a joint TB Research Network^11^ in 2016. Indian Prime Minister Modi recently announced a plan to end TB in India by 2025,^12^ and India has added $740 million to its national TB programme, roughly *quintupling* its investment.^13^ The Chinese First Lady is a TB global champion^14^ and, in 2017, Russian President Putin^15^ opened the WHO Global Ministerial Conference on Ending TB,^16^ publicly committing Russia to the global fight.

However, in spite of these political commitments, the fragmentation of the global TB market across multiple less lucrative markets, combined with scientific risk, deters sustained private sector investment in R&D. Because these high burden countries lack an effective mechanism to ‘pool’ their burden and resources—and thus signal the existence of an attractive, commercial market—there remains no cost-effective way for any individual country to address its disease burden through medical innovation.

## Our proposal: the market-driven, value-based advance commitment

With the right mechanism, we can leverage today’s political commitment to transform the landscape of TB innovation—from a struggling, donor-dependent ecosystem to a vibrant, country-driven marketplace delivering new treatments at affordable prices. We propose a new innovation model—the market-driven, value-based advance commitment (MVAC)^17^—that creates and guarantees a market for new TB drugs if and when innovators launch a breakthrough TB regimen. We define the latter as a short, universal regimen which tackles different types of TB, including drug-resistant strains.

The MVAC draws on Gavi’s Advanced Market Commitment (AMC)^18^ for pneumococcal vaccine.^19^ Like the AMC, the MVAC includes an advance commitment to purchase a product, if it materialises, at a pre-agreed price-volume combination (which results in the agreed market value.) Key differentiating factors, outlined in table 1, include these:

1. Use of health technology assessment (HTA) ensures prices are affordable and reflect value for payers;
2. Countries are not required to either pool or set aside funds until the regimen receives marketing authorisation; in the interim, they can use their healthcare budgets to buy products and services rather than fund R&D directly. Countries only pay for products they buy and receive.
3. Most importantly, the initiative is country-driven (vs donor-driven). Countries affected by TB would make advanced purchase commitments based on the local value of a universal TB regimen. These commitments are subsequently guaranteed by a multilateral development bank (MDB) intermediary, with ultimate accountability (to the MDB) based on countries sovereign credit-worthiness.
4. The poorest (low-income) countries would not be part of the MVAC; however, the contractual arrangement with the innovator would ensure they receive cost-plus access. The innovator could opt to licence the product to a generics manufacturer, potentially facilitated by the Medicines Patent Pool.^20^

**Table 1:** Leveraging the lessons learnt from previous AMCs to new models

Key factor	AMC pilot for pneumococcal vaccines	MVAC for TB
**Time frame for meeting the TPP**	Short, as products were in *late stage* of development	Long, as potential candidates are in *pre-clinical/early stage* of development
**TPP**	Product specifications defined by WHO experts including minimal characteristics to get reward	Product specifications defined by country payers, drawing on expert advice. Expert group to decide (as part of the governance) the minimum characteristics to get some reward. There are proposals around the minimum TPP, but these need to be reviewed and endorsed by the technical committee and ultimately by the countries.
**Price**	Initial AMC price (paid by donors to recover manufacturing investment) of $7, subsequently reduced to a tail price of $3.50, set at an estimate of the marginal cost of production. The $3.50 became the minimum price.	Price based on health technology assessment (HTA) value assessment of the TPP and on local ability to pay of BRICS. Different prices in different countries. Prices adjusted to reflect percentage of TPP met in practice by the products.
**Competition**	Non-exclusive scheme to cover first-generation and second-generation products Initial contract not to take all of the commitment Companies could compete on price and quality Effectively, however, rewarded two companies	Non-exclusive scheme to cover first-generation and second-generation products Companies can in principle compete on price and quality; however, complexity of meeting TPP means combinations are likely and competition unlikely.
**Countries it is designed for**	Designed to engage donor countries	All except HICs, with a focus on large MICs, but in particular countries transitioning away from aid; TB burden concentrated in large MICs and low-income countries
**Governance**	WHO experts defined the TPP Gavi served as secretariat and supported eligible countries to purchase the product The World Bank guaranteed the AMC fund UNICEF managed the supply agreements	Global secretariat (to be determined) and decision-making function on key scheme elements Advisory/expert committee (with MICs, global TB and HTA experts, donors, other stakeholders) to provide recommendations on the extent to which the new product meets the TPP
**Role of companies (developers and/or manufacturers**)	Enter the AMC Registered Manufacturers Agreement Scale up manufacturing capacity to meet Gavi-eligible countries’ demand for 10 years	Register interest at an early stage Develop and submit regulatory and HTA dossiers for the new product Commit to developing manufacturing capacity for the agreed period of time and price Show willingness to engage in a commercial agreement involving post-launch evidence collection
**Who bears the risk?**	Manufacturer bore R&D and manufacturing risk, donor bore volume risk	Multilateral development banks (MDBs) underwrite, companies bear R&D risk, countries bear volume risk (ie, commit to buying a certain value of the product)
**Role of donors**	AMC definition and governance (WHO, Gavi, UNICEF) Price top-up to reward innovation (global donors)	Facilitate scheme establishment Help mobilise political support for the proposal Potentially help cover costs for MVAC secretariat; subsidise or cover commitment fees for MDB guarantees; provide research grant funding for BRICS research bodies
**Role of LMICs**	Originally expected to contribute with a co-pay as a share of the tail price but in practice this has been met by global donors	Actively involved in the definition of the scheme Committing to pay a predefined price for a predefined volume based on their budget constraints and value offered by the prospective intervention(s)
**Role of financing intermediaries**	Donors guaranteed funding to Gavi. No intermediary.	Potential role for an MDB to provide loan financing to assist in guaranteeing the recipient commitments

AMC, Advanced Market Commitment; BRICS, Brazil, Russia, India, China and South Africa; HICs, high-income countries; LMICs, low- and middle-income countries; MICs, middle-income countries; MVAC, market-driven, value-based advance commitment; R&D, research and development; TB, tuberculosis; TPP, target product profile.

**Figure 2:**
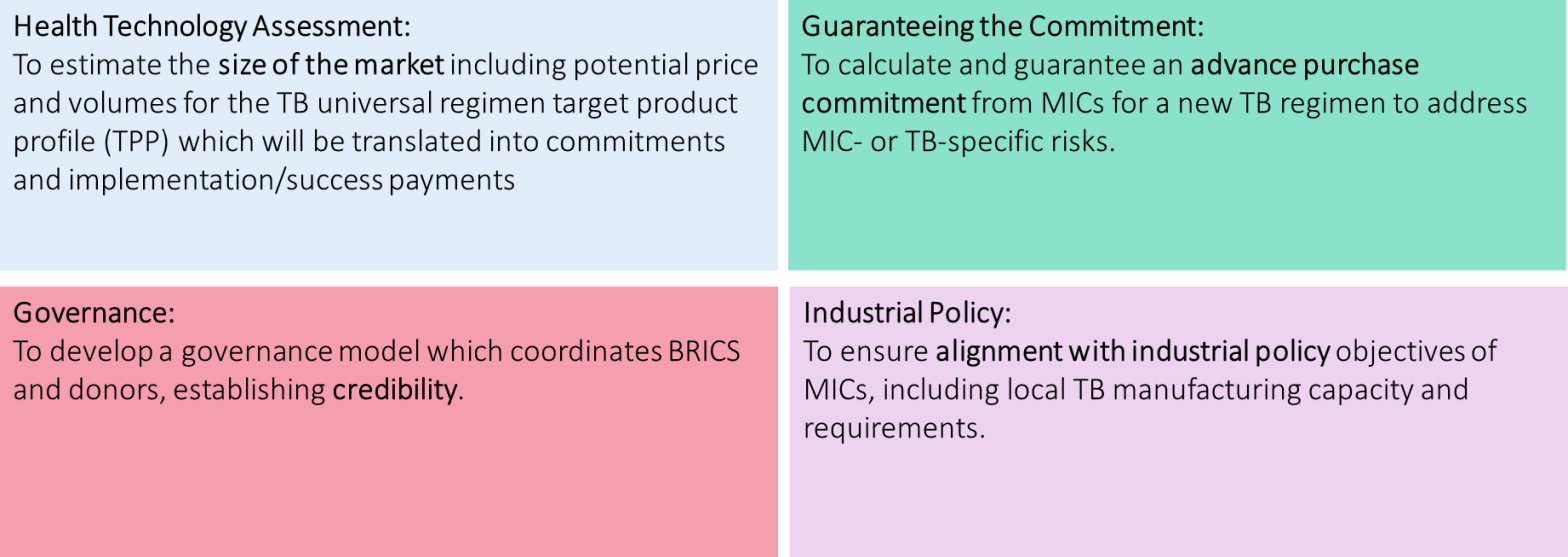
Market-driven, value-based advance commitment. SDG, Sustainable Development Goal; TB, tuberculosis.

Below we describe the MVAC’s four core elements as illustrated in figure 2.

### Element 1: HTA to support coverage decisions from the payer’s perspective

In 2014, the World Health Assembly endorsed HTA to help countries prioritise healthcare spending for universal health coverage. Low- and middle-income countries (LMICs) increasingly draw on HTA evidence when deciding whether new products should be adopted and/or eligible for public subsidy or reimbursement. HTA has also been applied by product developers early in the development process to assess pricing options and inform investment decisions.

For the MVAC, we propose to merge these two applications of HTA. We used well-validated TB epidemiology and health economic models^21^ to predict the likely value of a TB regimen in a sample of high-burden countries; this value assessment serves as the starting point for a price and volume commitment. Our HTA model is based on local country affordability thresholds, calibrated against future budgets; we also imagine a scenario where many new TB technologies (eg, TB vaccines, short regimens, new diagnostic tests) and policies (eg, aggressive private sector engagement in TB treatment in India) will be introduced, inflecting the comparator standard of care and therefore the value of the breakthrough regimen

We define the TB breakthrough innovation based on a single cross-country target product profile (TPP), previously developed through a WHO collaborative process, to tackle both drug resistance and the need for a shorter regimen. However, even a breakthrough regimen may not meet the entire TPP wish list, hence health gains need to be assessed on a country-by-country basis. Countries would need, however, to collectively define a minimum acceptable TPP to trigger the MVAC commitment; above the minimum threshold, more effective products would create additional value, and therefore generate a higher price and volume commitment.

### Element 2: guaranteeing the commitment

MIC purchase commitments can only prompt industry investment if they are perceived as highly credible. A commitment guarantee—underwritten by a financial intermediary—would help ensure that MICs credibly signal their demand and willingness to pay. The size of the commitment guarantees could vary depending on how many MICs were willing to participate.

To guarantee countries’ purchase commitments, countries would leverage their own sovereign creditworthiness—intermediated through a AAA-rated intermediary guarantor such as a MDB—to underwrite the advance commitments. After the drug comes to market, the country would have (say) 10 years to fulfil the entirety of its purchase commitment by purchasing drugs directly from the originator company or a local authorised licensee. If a commitment balance remains at the end of the 10-year window—that is, if a country were to partially or fully renege on its purchase commitment—the remaining balance would convert to a loan by the MDB, subject to repayment by the commitment-making country under pre-agreed terms. The remaining drug purchase commitment would be honoured by the MDB on behalf of the country, and the drugs would be supplied for the country to use as it thought appropriate. Note that no funds need be set aside by national payers prior to the launch of the product; at launch, countries would purchase the eligible product through standard budgetary, procurement and contractual mechanisms at a cost-effective and locally affordable price.

### Element 3: governance with national governments and local civil society in the driving seat

The MVAC is a vehicle for multinational and multi-stakeholder cooperation; ultimately, its structure and operations must be owned and governed by participating countries’ governments and civil society, in partnership with trusted global experts, institutional stakeholders and local and multinational industry. A Board of participating country governments, advised and informed by an HTA Technical Advisory Committee comprised of civil society, experts, patient advocates and other stakeholders, would hold ultimate decision-making authority. In turn, the Board would delegate day-to-day operations—for example, commissioning HTA models and analyses, preparing contracts and facilitating negotiations—to a permanent secretariat. This body would work hand-in-hand with existing or budding HTA/priority-setting institutions in participating countries.

Based on a needs’ assessment and broad consultation, we identified a World Bank trust fund as the best fit to host an MVAC secretariat. The World Bank is a credible multilateral institution—both for potential industry partners and for MICs, which already participate in institutional governance and could oversee a dedicated trust fund. The trust fund model is widely used to steward development resources and is well-trusted by the donors who might subsidise the secretariat’s operational costs. It can provide predictable multi-year funding—potentially using a single up-front investment to finance the MVAC secretariat over the entirety of its long-term life cycle.

### Element 4: industrial policy

Most BRICS countries have industrial strategies to support domestic companies, including within the pharmaceutical sector; these can include requirements for localisation, local manufacturing and/or clinical development partnerships. A successful MVAC innovator company could be expected to meet countries’ industrial policy requirements by, for example, licensing production to local manufacturers. Given high overall expected volumes, technology transfer models and license agreements between multinational developers and local manufacturing companies could help secure long-term supply; in so doing, it would be essential to ensure high quality and avoid unnecessary duplication.

Our approach respects both affordability considerations and intellectual property (IP) by moving price negotiations further upstream. Rather than waiting for a lifesaving product to (hopefully) come to market and then attempting to secure an affordable price—which may include either the threat of or application of compulsory licensing—MVAC country governments and industry will agree to a locally affordable but still profitable price *before the drug is developed*, simultaneously ensuring that industry will make the requisite investments to bring the product to market and that the product will be accessible to all who need it once launched. As a result, participating countries will not need to use Trade-Related Aspects of Intellectual Property Rights (TRIPS) flexibilities for this particular product and will agree to respect the originator IP rights, so long as the IP-holder offers the product at the pre-agreed affordable price. Ability to use TRIPS flexibilities remain in place for non-participating countries, and for all products not covered by an explicit MVAC agreement. To ensure that critical breakthrough treatments are not lost in the event that a developer should fail to follow through on the terms of MVAC, the agreement may include step-in-rights, allowing participating governments and the Secretariat to make the product available to MICs and LICs, respectively, as per the terms of the agreement.

## A work in progress

We have developed this proposal in close consultation with a broad range of stakeholders including civil society, international bodies, industry and national governments. We shared a draft of our thinking publicly in March 2019,^22^ sparking further discussion and collaborative problem-solving, and we continue to welcome constructive dialogue and engagement.

We do not have all the answers. Some key unresolved questions include: How will this model interface with other arrangements, such as the Life Prize? Can we encourage investment from small and medium enterprises—not just large multinational corporations? Will national payers agree to a differential pricing approach, potentially paying more than other countries if they reap higher value? Can we agree to put aside (for now) differences of opinion about intellectual property and de-linkage if we can ensure we achieve the key shared goal: access to lifesaving innovation at affordable prices?^23^

## We cannot afford inaction

Beyond addressing the immediate challenge of TB, our proposal^17^ offers a roadmap to leverage private-sector investment for LMIC health priorities. Through the MVAC, countries can strengthen their own technical HTA capacity to shape pharmaceutical policy and help secure affordable prices, as national health schemes in the UK, Australian and elsewhere have been doing for years. A value-based advance market commitment, supported by a multilateral development bank guarantee, can build trust between the demand and supply sides; this ‘bridging’ mechanism can help transition to a more functional relationship between industry and country governments that incentivises private-sector R&D investment into the diseases of the poor and vulnerable.

With 1.6 million people dying from TB each year, and few private sector resources going toward LMIC country disease priorities, we desperately need a new approach to health R&D and with an estimated $2.5 trillion financing gap to achieve the Sustainable Development Goals, business as usual means we will fail to achieve global health and development targets. We call on the global community and MIC leaders to take bold action and support and secure MVAC as a tool we need for the journey to build a TB-free world.
